# TRP Channels in Skin Biology and Pathophysiology

**DOI:** 10.3390/ph9040077

**Published:** 2016-12-14

**Authors:** Michael J. Caterina, Zixuan Pang

**Affiliations:** Departments of Neurosurgery, Biological Chemistry and Neuroscience, Neurosurgery Pain Research Institute, Johns Hopkins School of Medicine, 725 N. Wolfe St., Baltimore, MD 21205, USA; punfilict@gmail.com

**Keywords:** transient receptor potential, skin, pain, itch, dermatitis, epidermis

## Abstract

Ion channels of the Transient Receptor Potential (TRP) family mediate the influx of monovalent and/or divalent cations into cells in response to a host of chemical or physical stimuli. In the skin, TRP channels are expressed in many cell types, including keratinocytes, sensory neurons, melanocytes, and immune/inflammatory cells. Within these diverse cell types, TRP channels participate in physiological processes ranging from sensation to skin homeostasis. In addition, there is a growing body of evidence implicating abnormal TRP channel function, as a product of excessive or deficient channel activity, in pathological skin conditions such as chronic pain and itch, dermatitis, vitiligo, alopecia, wound healing, skin carcinogenesis, and skin barrier compromise. These diverse functions, coupled with the fact that many TRP channels possess pharmacologically accessible sites, make this family of proteins appealing therapeutic targets for skin disorders.

## 1. Introduction to TRP Channels

Transient Receptor Potential (TRP) channels constitute a large family of ion channels expressed across vertebrate and invertebrate animal species. Mammals express at least 28 different TRP channels that can be divided into six subfamilies, based on their primary amino acid structures: TRPA (ankyrin), TRPC (canonical), TRPM (melastatin), TRPML (mucolipin), TRPP (polycystin) and TRPV (vanilloid). [1,2,3]. TRP channels are widely distributed across tissues, such that every cell in the body likely expresses one or more subtypes. Furthermore, TRP channels can be gated by an astonishingly diverse array of physical and chemical stimuli, ranging from ions and small molecules to heat, cold, and mechanical force [1,2,3]. Consequently, TRP channels are important for many aspects of health and disease [4]. 

**Figure 1:**
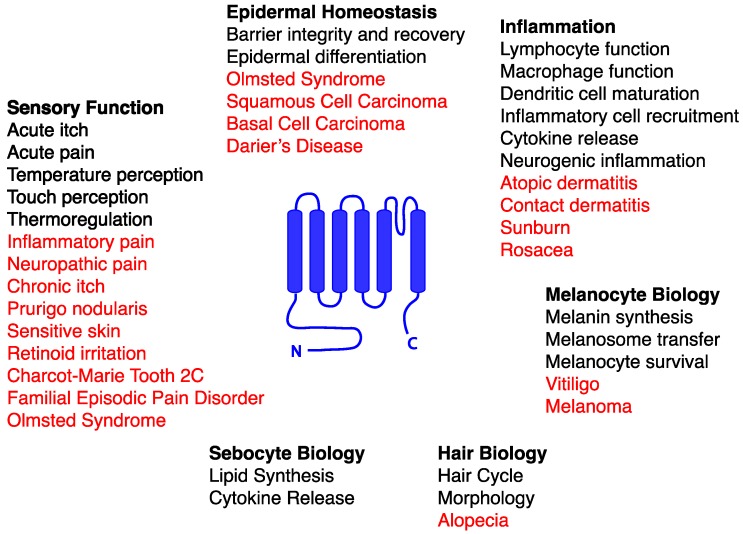
Summary of physiological (black) and pathophysiological (red) processes to which Transient Receptor Potential (TRP) channels contribute in various skin cell types. Topological diagram of a TRP channel subunit is shown at center, with amino (N) and carboxyl (C) termini labeled. Extracellular domain is at top.

Functional TRP channels consist of four subunits surrounding a central channel pore. In most cases, TRP channels are homotetramers of a given subunit, while in others, subunits of two different TRP channel subtypes contribute to a heterotetrameric channel. Although there are some exceptions, as described later in this chapter, each TRP channel subunit possesses six transmembrane domains, interconnected by relatively short loops, plus a relatively long N terminal domain and a somewhat shorter C terminal domain, with the latter two both extending into the cytoplasm (Figure 1). In some, but not all subfamilies, the N terminal domain contains multiple copies of a motif known as the ankyrin repeat that contributes to channel assembly and gating [2]. Recent cryo-electron microscopy studies of TRPV [5,6,7,8,9] and TRPA [10] family members have provided a high-resolution view of the structures of these channels, as well as insights into how their pores can be gated by their respective activators. All known TRP channels are selective for cations, with little or no anion permeability. However, their relative selectivity among cations can vary. Whereas most TRP channels are so-called nonselective cation channels that show permeability to both monovalent cations such as sodium and divalent cations such as calcium and magnesium, a few are highly selective for either calcium or sodium ions [3].

Like other tissues, the skin expresses an abundance of TRP channel subtypes that significantly affect its development, integrity, and function under healthy conditions and in disease states [11,12]. There are a number of fundamental mechanisms, described in greater detail throughout this review, through which this occurs. Most notably, the ability of many TRP channels to mediate calcium influx into cutaneous neurons, keratinocytes, melanocytes, or immune cells provides a mechanism by which these channels influence cellular proliferation, differentiation, secretion of paracrine/autocrine factors, cytotoxicity, cell migration, and a host of other processes relevant to skin health and disease. Calcium is of particular importance to keratinocytes, since the skin exhibits a gradient of extracellular calcium from relatively low levels in the basal epidermis to relatively high levels in more mature epidermal layers. This gradient, in part, helps to drive the progressive differentiation of epidermal keratinocytes as they are displaced apically by proliferating basal cells [13]. Dysregulation of calcium homeostasis, as occurs in conditions such as Hailey-Hailey disease [14] or Darier’s disease [15], results in abnormal epidermal differentiation, keratosis, poor adhesion between keratinocytes, and other abnormalities. Another important output of TRP channels is membrane depolarization. In cutaneous sensory neurons, TRP channel-mediated depolarization in response to a host of chemical, thermal, and mechanical stimuli triggers action potential firing, eventually leading to sensations of temperature, pain, or itch [16]. The effects of TRP channel-mediated depolarization, moreover, are not confined to neurons, since membrane depolarization in nonexcitable cells like keratinocytes or lymphocytes can affect cellular processes such as ATP release [17] or calcium flux through Orai family channels [18]. Finally, a growing list of protein interactors has been identified for TRP channels, further expanding the potential signaling repertoire of these molecules [19,20]. In this chapter, we will provide an overview of TRP channel expression in various cell types in the skin, as well as the physiological and pathological cutaneous processes to which they contribute. A summary of these diverse processes is presented in Figure 1, highlighting multiple opportunities for therapeutic targeting of TRP channels in skin.

## 2. Contributions of TRP Channels to Skin Biology and Pathophysiology

### 2.1. TRPC Channels and Skin

#### 2.1.1. TRPC Channels and Keratinocyte Differentiation

Expression of nearly every TRPC channel subtype, including TRPC1, TRPC3, TRPC4, TRPC5, and TRPC6, has been reported in human keratinocytes. TRPC1 and TRPC4 are both upregulated during keratinocyte differentiation in vitro. This expression level change appears to be functionally important, since knockdown of either TRPC1 or TRPC4 in keratinocytes in vitro was shown to reduce the level of store-operated calcium entry and to inhibit keratinocyte differentiation [21,22]. TRPC6 has also been shown to be required for normal human keratinocyte differentiation in vitro [23]. In addition, small molecules of the triterpine family have been shown to promote keratinocyte differentiation both in vitro and in vivo through a mechanism that appears to involve TRPC6 [24].

#### 2.1.2. TRPC Channels in Darier’s Disease

Darier’s Disease, also known as Darier-White disease, is a rare autosomal dominant dermatological disease initially discovered by the French dermatologist Ferdinard-Jean Darier in 1889. It is characterized by keratotic papules that may occur throughout the body [25]. Darier’s Disease is caused by heterozygous loss of the endoplasmic reticulum calcium pump protein SERCA2b, which is encoded by the gene ATP2A2 located on 12q23-24.1 [26]. In addition to skin papules, patients with this disorder often suffer from neuropsychiatric symptoms [27]. There have also been reports of bone cysts [28]. Because SERCA2b is the only SERCA pump expressed in keratinocytes, this defect results in failure to sequester calcium in the ER lumen and the consequent accumulation of excess cytoplasmic calcium. Interestingly, examination of TRP channel expression in keratinocytes from heterozygous SERCA knockout mice revealed a compensatory upregulation of TRPC1 [29]. Although this change might a priori be predicted to worsen calcium overload in these cells, it was found that TRPC1 upregulation somehow enhanced keratinocyte resistance to apoptosis [29]. Whether compensatory TRPC1 expression ameliorates or exacerbates the Darier’s Disease phenotype in intact skin, however, remains unclear. It also remains to be determined whether other syndromic features of Darier’s disease involve alterations in TRP channel-mediated calcium signaling in skeletal or nervous tissues similar to those proposed for keratinocytes.

#### 2.1.3. TRPC Channels and Sensory Function

There are numerous connections between TRPC channels and cutaneous sensory function. Although TRPC channels are expressed in most cell types, TRPC1, TRPC3, TRPC5 and TRPC6 are the subfamily members characterized most extensively in sensory neurons. Based on pharmacological studies with the relatively nonselective TRPC channel antagonist, SKF-96365, TRPC channels might also contribute to pain and hyperalgesia caused by the bee venom component, mellitin [30]. TRPC3 is expressed in small- to medium-diameter nociceptors [31,32,33], and has been shown to be important for store-operated calcium entry responses downstream of G protein-coupled receptors for purines and proteases [32], providing a potential mechanism for the involvement of this channel in some forms of inflammatory hyperalgesia. TRPC3 has further been implicated in nociceptor activation by IgG immune complexes and thus might be a contributor to pain in allergic contact dermatitis [34]. It has also been shown that TRPC1 and TRPC6, which are co-expressed in sensory neurons with TRPV4, facilitate hyperalgesic responses mediated by the latter channel, through mechanisms that have not been clearly resolved [31]. Further evidence for a role for TRPC1 in sensory function comes from the observation that this channel is required for normal mechanically evoked responses in two subpopulations of mechanosensory cutaneous neurons (Aδ-low threshold mechanoreceptors and slowly-adapting Aβ-low threshold mechanoreceptors) and for behavioral responses to very gentle mechanical stimuli [35]. Finally, TRPC5, which is also expressed in a subpopulation of peripheral sensory neurons that innervate the skin, can be activated by mildly cold temperatures. However, no obvious defects in cold detection were observed in mice lacking this channel [33].

### 2.2. TRPV1 and Skin

TRPV1 is among the most extensively characterized mammalian TRP channels. Upon gating, this channel produces multiple cellular signals, including membrane depolarization and an increase in cytoplasmic calcium. TRPV1 is expressed at the highest level in a subpopulation of peptidergic peripheral sensory neurons involved in the perception of pain. When overexpressed recombinantly in cell lines, TRPV1 can be activated by capsaicin, the major pungent ingredient in chili peppers, or by related chemical compounds that share a vanilloid chemical group, thus providing the “transient receptor vanilloid” subfamily its name [1]. TRPV1 can alternatively be activated by extracellular protons, by certain small lipophilic molecules, including endogenous cannabinoid lipids such as anandamide and N-arachidonoyl dopamine [1], or by a number of other chemical agonists such as 2-aminoethoxydiphenyl borate (2-APB), which had previously been recognized as a dose dependent activator and inhibitor of IP3 receptors and store-operated calcium channels [36]. In addition, TRPV1 was the first recognized molecular thermoreceptor, as it can be activated in the absence of chemical ligands by painfully hot temperatures (>42 °C) [1]. This polymodal chemo-thermo sensitivity likely accounts for the perception of “heat” experienced during consumption of chili peppers, and has led TRPV1 to receive substantial attention as a candidate target for pain control [37].

#### 2.2.1. TRPV1 in Pain, Itch, and Neurogenic Inflammation

Gene knockout studies in mice, as well as administration of TRPV1 antagonists to mice, rats, and humans, have confirmed roles for this channel in pain sensation. For example, mice lacking TRPV1 are insensitive to capsaicin and show partially diminished heat-evoked pain at baseline and a virtual absence of inflammation-induced thermal hyperalgesia [38,39]. Reductions in heat pain [40], inflammatory pain [41,42] and cancer pain [43,44] sensitivity have also been observed in rodents and/or human treated with TRPV1-selective antagonists. Furthermore, a number of phase I and II clinical trials of TRPV1 antagonists have been conducted to examine their potential analgesic utility [45,46]. Studies using TRPV1 antagonists have also provided evidence for a role of TRPV1 molecules located at the central terminals of primary sensory neurons in certain forms of mechanical hypersensitivity, though the cellular mechanisms underlying such roles remain to be elucidated [47].

Beyond the perception of pain, TRPV1 has been shown to participate in other neuronal functions relevant to the skin. For example, TRPV1 null mice exhibit reduced itch-related scratching behavior in response to interleukin 31 (IL31) [48] or histamine [49]. TRPV1 also facilitates the function of TRPV4 in itch perception [50]. In addition, capsaicin-induced neurogenic inflammation (i.e., inflammation produced by the neuronal release of vasoactive peptides) is suppressed in the absence of TRPV1 [1,51]. Therefore, the interaction of neuronally-expressed TRPV1 with the skin is bidirectional, involving both sensory and efferent activities.

#### 2.2.2. TRPV1 Expression in Keratinocytes

Aside from the functional importance of neuronally-expressed TRPV1, there is considerable evidence to suggest that TRPV1 may participate more directly in epidermal biology by virtue of its expression in keratinocytes. Evidence for such expression is strongest in human keratinocytes. Inoue et al. [52] first reported the existence of TRPV1-like immunoreactivity and TRPV1 mRNA in cultured human keratinocytes. They also showed that in these keratinocyte cell cultures, capsaicin or protons could evoke an influx of calcium that was inhibited by the TRPV1 antagonist, capsazepine. In contrast, whereas primary neonatal mouse keratinocytes and the mouse 308 keratinocyte cell line were shown to express TRPV1 mRNA, they did not respond to capsaicin in physiological assays [53,54]. Immunological, mRNA, and calcium imaging-based evidence for TRPV1 expression was also reported in the human HaCaT keratinocyte cell line [55]. In these cells, capsaicin could evoke the release of IL8 and prostaglandin E2 (PGE2) and upregulate the expression of cyclooxygenase 2 (COX2), providing evidence for potential proinflammatory activities. TRPV1 agonist-mediated calcium influx was also observed in human NHEK keratinocytes, and these responses, as well, could be blocked by either capsazepine or another TRPV1 antagonist, PAC-14028 [56]. Furthermore, heat shock was shown to trigger matrix metalloproteinase 1 (MMP-1) transcription in both HaCaT and NHK keratinocytes, in a manner apparently dependent on TRPV1 [57]. There is additional evidence that TRPV1 may be expressed in keratinocytes in intact healthy human skin. For example, Stander et al. [58] observed TRPV1-like immunoreactivity in keratinocytes of the stratum basalis and stratum granularis, as well as in dermal mast cells, hair follicles, sebaceous gland epithelial cells, and cutaneous sensory nerve terminals. A common problem with many human skin immunostaining studies is the absence of definitive controls for antibody specificity. However, the possibility of intrinsic cutaneous TRPV1 expression in that same study was corroborated by polymerase chain reaction detection of TRPV1 mRNA.

#### 2.2.3. TRPV1 in Epidermal Homeostasis and Dermatitis

Despite the challenges of definitive demonstration of TRPV1 expression in rodent keratinocytes, multiple studies in rodents have suggested TRPV1 involvement in epidermal barrier function and the regulation of dermatitis. In hairless mice, capsaicin delays epidermal barrier recovery following tape-stripping and the TRPV1 antagonist capsazepine accelerates such recovery [59]. A potential role for heat as a mediator of this effect arose indirectly from the observation that barrier recovery is accelerated at relatively warm skin temperatures (36 °C–40 °C), compared with cooler temperatures. Agonists of two other warmth-sensitive TRP channels found in keratinocytes, TRPV3 and TRPV4 (see below), failed to recapitulate the effects of heat. In a series of studies [56,60,61], a TRPV1 antagonist, PAC-14028, was shown to accelerate skin barrier recovery either following tape stripping or in two models of atopic dermatitis: *Dermatophagoides farina* (Df) challenge in a susceptible mouse strain and challenge with the small molecule hapten oxazolone in previously sensitized mice. PAC-14028 also reduced rises in serum immunoglobulins, skin thickening, mast cell degranulation, and scratching behavior following repetitive Df administration. However, the apparent direction of TRPV1 effects on epidermal homeostasis and dermatitis has not been uniform across studies. In one study, oxazolone induced ear edema was increased in mice lacking TRPV1 or in wild-type mice in which TRPV1-expressing neurons were desensitized with vanilloid compounds [62]. The authors of that study postulated that anti-inflammatory agents released by TRPV1 expressing neurons accounted for the apparently anti-dermatitic effects of this channel. Although the reasons for discrepancies among these studies remain unclear, they might include the use of immunologically distinct mouse strains or off-target effects of some of the TRPV1 modulating agents used. Furthermore, a definitive dissection of the functions of neuronal vs. non-neuronal TRPV1 function in these models remains to be performed.

#### 2.2.4. TRPV1 and Ultraviolet Radiation

Lee et al. [63] demonstrated that ultraviolet B (UVB) light could induce a calcium influx in HaCaT cells that was sensitive to TRPV1 antagonists, as well as an increase in MMP1 expression that was suppressed by TRPV1 antagonists and TRPV1 siRNA knockdown and facilitated by capsaicin. This study also reported a UVB-induced increase in TRPV1 western blot signal in HaCaT cells and a UVB induced increase in TRPV1 immunostaining in human skin. Based on their findings, the authors of this study speculated on a potential role for TRPV1 in UVB induced skin aging. Consistent with human keratinocyte studies, in hairless mice, the TRPV1 antagonist, iodo-resiniferatoxin, could suppress UVB induced skin thickening and expression of MMP, COX2, and p53 [64]. However, whether that effect was keratinocyte-intrinsic or neuronally mediated was again not definitively established.

#### 2.2.5. TRPV1 Epidermal Upregulation in Human Skin Diseases 

Elevations in either epidermal TRPV1-like immunostaining or skin TRPV1 mRNA expression have been reported in several different human skin diseases, including prurigo nodularis [58], rosacea [65], and herpes zoster infection [66]. In addition, there may be a link between TRPV1 and sensitive skin, as defined by augmented sensitivity in the lactic acid stinging test [67]. TRPV1 mRNA and keratinocyte TRPV1-like immunostaining were elevated in patients with positive responses in this psychophysical test [67]. These patients are presumed to have either an impaired skin barrier and/or alterations in their neurovascular responsiveness [67]. Interestingly, TRPV1 immunoreactivity was lower in individuals with darker skin, suggesting that melanin might interfere with those factors that promote TRPV1 upregulation [67]. Perhaps related to these findings, the skin irritant phenoxyethanol was found to increase calcium influx into HaCaT cells in a manner inhibitable by TRPV1 antagonists [68]. Furthermore, it has been shown that retinoids evoke pain behavior in rodents by acting at TRPV1, presumably in neurons. This might explain the burning sensation reported for these compounds in humans [69].

#### 2.2.6. TRPV1 and Skin Cancer

Some studies have provided evidence suggestive of a connection between TRPV1 and skin cancer. However, these findings have often been indirect and relied upon pharmacological tools. For example, capsaicin was shown to be co-carcinogenic in rodent skin [70] and the TRPV1 antagonist AMG9810 was shown to act as a tumor promoter [71] However, in the former study, the effects of capsaicin turned out to be mediated by EGFR signaling, independent of TRPV1, while in the latter study, the TRPV1 dependence of the AMG9810 effects, which also somehow involved EGFR signaling, was not directly addressed. Thus, no role for TRPV1 in skin cancer has yet been definitively established.

#### 2.2.7. TRPV1 in Skin Appendages

In human hair follicles, TRPV1-like immunoreactivity was observed in specific epithelial subcompartments, including the outer root sheath and hair matrix [58,72]. In organ cultures of these hair follicles, activation of TRPV1 suppressed epithelial proliferation and hair shaft elongation and promoted hair follicle regression [72]. TRPV1-like immunoreactivity has also been reported in epithelial compartments of mouse hair follicles, and examination of hair cycle in TRPV1 knockout mice revealed a delayed catagen phase [73].

There is also evidence for TRPV1 expression in human sebocytes, the major constituents of cutaneous sebaceous glands. In these cells, TRPV1 stimulation with capsaicin suppresses lipid synthesis and the release of proinflammatory cytokines [74].

### 2.3. TRPV2 and Skin

TRPV2 was originally discovered as a calcium-permeable channel that could be regulated by insulin-like growth factor I signaling [75], and in parallel, as a channel that could be activated by extremely high temperatures (>52 °C) [76]. TRPV2 was subsequently found to be capable of being activated by a range of stimuli, including PI3 kinase signaling [77], certain cannabinoid compounds (Δ9-tetrahydrocannabinol, cannabidiol) [78], probenecid [79], 2-APB [36], hypoosmolarity [80], and mechanical cell stretch [81]. In the skin, TRPV2 is most highly expressed in two categories of cells: sensory neurons and immune/inflammatory cells.

#### 2.3.1. TRPV2 and Sensory Function

The contribution of TRPV2 to sensory function remains enigmatic. Although rodent TRPV2 has been shown to be activated by heat [76], mice lacking TRPV2 showed no detectable defects in noxious heat sensation, even if the potentially confounding activity of TRPV1 was eliminated [82]. Furthermore, heat sensitivity appears not to be conserved in human TRPV2 [83]. There is evidence that TRPV2 plays a role in neurite outgrowth, particularly in response to mechanical stretch of neurons [81] or nerve growth factor stimulation [84]. Whether this has implications for nerve regeneration in the skin has yet to be explored.

#### 2.3.2. TRPV2 and Immune Cell Function

TRPV2 expression has been reported in a range of immune/inflammatory cell types, including macrophages, mast cells, natural killer cells, dendritic cells and lymphocytes [85]. Within these cell types, TRPV2 has been implicated in regulating diverse functions that include cytokine release [86], chemotaxis [87,88], phagocytosis [87], endocytosis [89], inflammasome activity [90], and podosome assembly [91]. In human skin, elevated TRPV2-like immunoreactivity was observed in both macrophages and mast cells in the setting of rosacea [65]. Aberrant TRPV2 expression has also been reported in hematological tumors and cell lines, including those derived from mantle cell lymphoma, multiple myeloma, Burkitt lymphoma, acute myeloid leukemia, and myelodysplastic syndrome [92,93].

### 2.4. TRPV3 and Skin

The cloning of TRPV3 was reported almost simultaneously by three groups [94,95,96]. TRPV3 mRNA and protein were shown to be expressed prominently in skin keratinocytes [94,96]. Like several other TRPV channels, TRPV3 can be activated by thermal or chemical stimuli. TRPV3 exhibits a temperature-dependent rise in activity at temperatures exceeding 33 °C–39 °C, with the specific apparent threshold varying among studies [94,95,96]. Reported chemical agonists of TRPV3 include 2-APB [36,97], farnesyl Pyrophosphate [98], and various plant-derived compounds, including camphor [99], carvacrol, eugenol, and thymol [100]. TRPV3 responses agonist stimulation can be further potentiated by several factors, including unsaturated fatty acids [101], repetitive heat stimulation [96], or cholesterol [102]. Conversely, factors that suppress TRPV3 activity include oxygen-dependent hydroxylation of TRPV3 by Factor-inhibiting-hypoxia inducible factor [103].

#### 2.4.1. TRPV3 and Cutaneous Temperature Sensation

The robust expression of TRPV3 in keratinocytes, as well as this channel’s heat-responsiveness, led a number of labs to investigate potential roles for this channel in skin temperature sensation. In vitro experiments in both the mouse 308 keratinocyte cell line and primary keratinocytes demonstrated warmth-evoked currents that resembled those mediated by recombinantly expressed TRPV3 [54]. Accordingly, these TRPV3-like currents were lost in keratinocytes isolated from TRPV3 knock out mice [99]. In contrast, TRPV3 protein proved difficult to detect in sensory neurons [99]. Together, these findings suggested that TRPV3 might participate in an “indirect” mechanism of heat sensation involving skin keratinocyte communication with sensory neurons. Consistent with this notion, TRPV3 was shown to mediate the heat-evoked release of a number of mediators with the capability to influence neuronal activity. For example, in keratinocyte-sensory neuron cocultures, heat activation was reported to produce a rise in keratinocyte calcium levels, followed by a delayed calcium influx response in the neurons, an effect that was apparently mediated by ATP signaling and dependent on keratinocyte TRPV3 [104]. It was also demonstrated that in keratinocytes, heat evokes the release of nitric oxide through a mechanism that is independent of nitric oxide synthase, and that this response is dependent on TRPV3 [105]. Similarly, keratinocytes cultured from mice overexpressing TRPV3 selectively in keratinocytes showed augmented release of prostaglandin E2 (PGE2) in response to both heat and 2-APB stimulation [106]. Together, these findings provided evidence that TRPV3 may serve as an important role in keratinocyte-neuron communication.

At the whole-animal level, there is also evidence that TRPV3 might contribute, under some circumstances, to heat perception. The original description of TRPV3 knockout mice reported that these mice exhibited deficits in both thermal preference behavior and heat-evoked nociception [99]. However, subsequent studies of these behaviors in TRPV3 knockout mice on more homogeneous genetic backgrounds yielded a more complex picture. Although the absence of TRPV3 on a pure C57Bl6 background produced no detectable change in thermally-evoked behavior, in two separate studies, TRPV3 knockout on the 129/S6 [107], or 129S1/SvImJ [105] backgrounds resulted in subtle alterations in thermal preference behavior. Interestingly, in one of these studies [105], the TRPV3 phenotype was sex dependent, occurring only in females. It should be noted that all these observations were made in global TRPV3 knockout mice, in which the channel was absent from all tissues throughout life. It is possible that a more definitive conclusion about the role of TRPV3 might be obtained from analysis of inducible keratinocyte- or neuron-selective TRPV3 knockout in adults. Mice lacking both TRPV3 and TRPV4 (see below) exhibited virtually normal thermal preference behavior and only a slight deficit in heat-evoked nociception [107]. However, in another study, it was demonstrated that while in mice lacking TRPV1 alone, a dynamic hotplate evoked an unusual escape behavior, this phenotype was abolished in mice lacking both TRPV1 and TRPV3 [108]. Thus, while TRPV3 might be a partial contributor to heat sensation under specific conditions, these genetic studies suggest that it is not a major participant in this process.

#### 2.4.2. TRPV3 and Epidermal Homeostasis and Hair Development

A number of studies have implicated TRPV3 in epidermal homeostasis and hair growth. Global TRPV3 knockout in mice was originally reported to produce transient alterations in abdominal hair morphology [99]. Subsequently, selective knockout of TRPV3 in mouse keratinocytes was found to produce several skin phenotypes, including perinatal skin barrier defects and abnormal epidermal maturation that appeared to resolve spontaneously and curly body hair and whiskers [109]. Similarities to reported effects of perturbations in EGFR signaling led the investigators of this latter paper to explore a potential relationship between these two signaling proteins. Indeed, they found that activation of TRPV3 in keratinocytes triggers the protease-mediated shedding of the EGFR ligand, TGF-α. They also found that TRPV3 could promote transglutaminase activity in keratinocytes, providing a potential explanation for the skin barrier defects in TRPV3 knockout mice. At least one human study also implicated TRPV3 in the regulation of hair growth [110]. These authors showed that application of the TRPV3 agonists eugenol or 2-APB to either cultured human hair follicles or outer root sheath (ORS) keratinocytes, produced a dose-dependent suppression of proliferation and induction of apoptosis in both systems.

#### 2.4.3. TRPV3 and Skin Pathology

There is also a growing body of evidence suggesting that TRPV3 is a key contributor to epidermal homeostasis and skin sensory function in certain pathological conditions. For example, although published studies are limited, there are reports in the patent literature that TRPV3 antagonists can suppress certain forms of mechanical hypersensitivity in rats after nerve injury [111]. In addition, it was reported that, in the mouse acetone-ether-water model of chronic dry skin, the genetic absence of TRPV3 resulted in reduced scratching behavior [112]. TRPV3-like immunoreactivity was also noted to be upregulated in the skin of breast surgery patients who reported postsurgical pain [113]. Similarly, TRPV3 mRNA was found to be upregulated in keratinocytes derived from patients with hypertrophic post burn scars who also reported itching [114]. Moreover, mouse studies have linked TRPV3 to epithelial wound healing in skin and oral mucosa [105,115].

The most compelling association between TRPV3 and skin pathology has come from the study of naturally occurring mutations in the gene encoding this channel. The earliest indications of this link came from rodent studies, where two TRPV3 mutations at the same residue (Gly573Ser and Gly573Cys, respectively) were found to be the causes of alopecia in two different rodent lines, the DS-Nh mouse and the WBN/Kob-Ht rat [116]. Further research by the same group [117] indicated that epidermal sheets derived from Ds-Nh mice exhibited elevated intracellular calcium levels. This finding is consistent with the subsequent finding that both the Gly573Ser and Gly 573Cys mutations confer a high level of constitutive activity on recombinant TRPV3 [118]. Additional examination of the in vivo consequences of these same TRPV3 mutations, resulted in additional skin phenotypes that included enhanced predilection towards evoked allergic contact dermatitis or, in some cases, spontaneous dermatitis [116,119]. The development of these dermatitis phenotypes was quite variable between studies and even within a study, between mice crossed onto different genetic backgrounds or housed under different conditions. This variable penetrance suggests the existence of both intrinsic and environmental modifiers of the dermatitis phenotype.

It is especially noteworthy that pathological gain-of-function mutations in TRPV3 are not confined to rodents. In 2012, whole-exome sequencing of six patients with Olmsted Syndrome revealed a TRPV3 mutation is strongly associated with this disease [120]. Olmsted Syndrome is a severe dermatological disease characterized by bilateral palmoplantar and periorificial keratoderma as well as severe itching or pain. Remarkably, the mutations discovered in Olmsted patients were homologous to those observed previously the Ds-Nh mice. The discovery of the link between Olmsted Syndrome and TRPV3 led to additional genetic studies in this condition. As a result, an increasing number of Olmsted patients were found to have TRPV3 mutations. Thus far, 10 TRPV3 mutations have been associated with approximately 20 Olmsted patients [121]. In addition, investigators recently discovered a gain-of-function mutation in a Chinese family that appears to be the cause of focal palmoplantar keratoderma [122] and another that was linked to another form of hereditary palmoplantar keratoderma without associated perioreficial lesions [121].

### 2.5. TRPV4 and Skin

TRPV4 was originally identified as a widely-expressed TRP channel that could be activated by changes in extracellular osmolarity [123,124,125]. Subsequently, it was shown that this channel could be activated by a range of physical and chemical stimuli, including cytochrome P450 metabolites of arachidonic acid [126], and warm temperatures [127]. Consistent with its expression pattern and wide range of activators, TRPV4 has been implicated in numerous processes in health and disease, many of which involve the skin.

#### 2.5.1. TRPV4 and Epidermal Barrier Function

TRPV4 is abundantly expressed in skin keratinocytes [127]. A role for this channel in epidermal barrier homeostasis comes from the observations that activation of this channel increases intracellular calcium in human keratinocytes and promotes cell-cell junction formation between these cells, and that knockdown of TRPV4 expression impairs development of high transepithelial resistance in cultured human keratinocytes [128]. Furthermore, warm temperatures and chemical agonists of TRPV4 accelerate barrier recovery in explanted human skin tissues after stratum corneum removal [129].

#### 2.5.2. TRPV4 and Skin Cancer

TRPV4 mRNA and immunohistochemical staining are reduced in premalignant skin lesions and in basal and squamous cell carcinomas [130]. Whether there is a functional role for TRPV4 in tumorigenesis has yet to be determined.

#### 2.5.3. TRPV4 and Sensory Function

Multiple studies have provided evidence for roles for TRPV4 in sensory processes. In some cases, these functions are attributable to keratinocyte-expressed TRPV4, while in others TRPV4 channels expressed in neurons may be the more relevant pool. TRPV4 has been shown to participate in osmotically-evoked pain behaviors [131,132] and also in acute mechanical nociception [133,134] and in mechanical hyperalgesia in certain models of inflammatory [135,136,137] and neuropathic [138,139,140] pain. In some chemotherapy induced neuropathy models, mechanical hyperalgesia appears to involve alterations in the interactions between TRPV4, α2β1 integrin, and src kinase [138]. Certain pro-algesic agents, such as agonists of the protease-activate receptor PAR2, also produce mechanical hyperalgesia through a process that involves TRPV4 [141]. TRPV4 has also been implicated in sunburn-associated hyperalgesia. TRPV4 mediates the release of the nociceptive/pruriceptive peptide, endothelin-1 from keratinocytes in response to UVB irradiation [142]. Moreover, mice lacking TRPV4 or treated with TRPV4 antagonists exhibited reduced UVB induced inflammation and mechanical and thermal hyperalgesia [142]. This effect is partially keratinocyte-autonomous, since it was observed in keratinocyte conditional TRPV4 knockout animals, and since TRPV4 mediates calcium entry into cultured mouse keratinocytes upon UVB exposure [142]. Consistent with these findings in mouse, TRPV4-like immunoreactivity was found to be elevated in human skin following UVB exposure [142].

There is a growing body of evidence implicating TRPV4 in itch perception. TRPV4 appears to play a role in itch perception in response to some, but not all pruritogens [143,144]. This is attributable at least in part to TRPV4 function in keratinocytes, where calcium influx through this channel triggers ERK phosphorylation. Scratching behaviors evoked by several pruritogens, including histamine, compound 48/80, and endothelin-1, but not that evoked by chloroquine, were partially suppressed in mice in which TRPV4 was deleted from keratinocytes [144]. However, contributions from sensory neuron-expressed TRPV4 to itch perception cannot be excluded. In both histaminergic and non-histaminergic itch, the contribution of TRPV4 appears to be facilitated by TRPV1 [50].

TRPV4 has also been implicated in innocuous warmth sensation, although the effect of TRPV4 knockout on this function is modest and condition-dependent. For example, whereas mice lacking TRPV4 were originally reported to show a shift in thermal preference on a thermal gradient towards slightly warmer temperatures [145], in later studies, as indicated above, mice lacking both TRPV4 and TRPV3 showed apparently normal thermal selection behavior, with only a slightly delayed withdrawal response to painful heat [107]. 

Numerous point mutations in TRPV4 have been shown to produce Charcot Marie Tooth disease type 2C [146,147,148]. Although not a skin disease per se, CMT2C is a sensorimotor degenerative disease that can include mild sensory loss.

### 2.6. TRPV6 and Skin

#### TRPV6 and Keratinocyte Differentiation

TRPV6, one of the most calcium-selective of mammalian TRP channels, is expressed in keratinocytes, where it plays important roles in epidermal differentiation [23]. Two different stimuli that are known to promote keratinocyte differentiation, elevations in extracellular calcium and 1, 25, dihydroxyvitamin D3, both upregulate transcription of TRPV6 [23]. This channel, in turn, mediates calcium influx to elevate basal intracellular levels [23]. As evidence of the importance of this process, in vitro siRNA silencing of TRPV6 suppresses the differentiation of keratinocytes in response to a calcium switch [23]. Moreover, one form of thermal spring water was shown to augment human keratinocyte differentiation in vitro through a mechanism that appeared to involve TRPV6 [149]. In addition, mice lacking TRPV6 exhibit an abnormally thin stratum corneum and a defective epidermal calcium gradient [150]. Although elevated TRPV6 expression has been associated with increased aggressiveness of prostate cancer [151], a potential link between TRPV6 and skin cancer has not been explored.

### 2.7. TRPA1 and Skin

TRPA1 is a nonselective cation channel that is abundantly expressed in a subpopulation of nociceptive sensory neurons, in addition to numerous other cell types. This channel is noteworthy for its direct and indirect responsiveness to an astoundingly diverse range of chemical, thermal, and mechanical stimuli. One large class of chemical TRPA1 activators includes electrophillic agents such as allyl isothiocyanate (mustard oil), cinnamaldehyde, acrolein, tear gas constituents, formaldehyde, and certain prostaglandins such as 15-deoxy-Δ12,14-prostaglandin J2. These electrophiles activate TRPA1 by covalent modification of specific cysteine residues located in the channel’s cytoplasmic N-terminus. Many non-electrophillic chemicals, including certain anesthetics (e.g., propophol, isofluorane, lidocaine), fenamate nonsteroidal anti-inflammatory drugs, cannabinoids (e.g., ∆(9)-Tetrahydrocannabinol ), cooling agents (e.g., icillin), and intracellular calcium ions can also activate TRPA1, presumably via more conventional ligand-receptor interactions. Its regulation by intracellular Ca^2+^ makes TRPA1 an effective integrator of other excitatory signaling pathways [152]. TRPA1 also exhibits a complex and species-specific pattern of thermosensitivity. In certain invertebrate and reptile species, TRPA1 can be activated directly or indirectly by warm temperatures and is essential for the detection of these temperatures in vivo [153,154,155]. Massive overexpression of TRPA1 in trigeminal sensory neurons likely accounts for the exquisite sensitivity of pit vipers to their warm prey [156]. In mammals, conversely, TRPA1 was originally reported to be activated by intense cold [157], a phenomenon that was recapitulated in some [158] but not all subsequent studies. Warm temperatures were shown to suppress and desensitize rat TRPA1 [159]. However, it was also recently shown that human TRPA1 exhibits a U-shaped temperature-response profile, with activation by both cold and heat. The complexity of this thermosensory behavior may in part be a consequence of strong TRPA1 regulation by modulating factors such as redox state [160]. TRPA1 proline hydroxylation analogously mediates changes in TRPA1 cold sensitivity in response to intracellular oxygen concentrations [161]. TRPA1 can also be activated in response to certain pathogen-associated molecular patterns and host-derived damage-associated molecular patterns. These effects can be either direct (e.g., lipopolysaccharide [162]) or indirect (e.g., let-7 miRNA acting via TLR7 [163]). Finally, TRPA1 has been implicated in mechanosensory processes [164,165,166]. However, this may reflect indirect mechanisms, rather than direct mechanical activation of the channel. Indeed, whereas classical mechanically-activated currents have not been reported in excised patches from cells expressing TRPA1, hypertonic stimuli can activate this channel in heterologous expression systems [167].

#### 2.7.1. TRPA1 and Cutaneous Pain Sensation

The complex pattern of direct and indirect response characteristics described above renders TRPA1 an active participant in a host of sensory and non-sensory processes in both health and disease, many of which involve the skin. For example, studies employing knockout mice or TRPA1 selective antagonists have revealed essential functions for TRPA1 in multiple aspects of cutaneous pain sensation, including pain evoked by electrophilic chemicals (e.g., mustard oil, formalin) or mechanical stimulation [165,168,169]. In the case of cold-evoked pain, the importance of TRPA1 is less evident under baseline conditions, but becomes exaggerated in the setting of tissue injury or inflammation [170]. TRPA1 is also a key participant in two common models of pathological chronic pain, streptozotocin-induced diabetic neuropathy [171] and chemotherapy-induced neuropathy [172]. At least part of the acute pain component in the former model is attributable to activation of TRPA1 by streptozotocin-generated peroxynitrite [173]. However, even later stages of neuropathic pain, including the loss of peripheral nerve fibers, are inhibited by the inhibition of TRPA1 [174]. Moreover, methylglyoxal, a metabolic byproduct of diabetic hyperglycemia, is an electrophillic TRPA1 activator [171]. In the case of chemotherapy-induced neuropathy, TRPA1 appears to be activated indirectly by platin-induced alteration of redox state [172]. Mutations or polymorphisms in TRPA1 have also been linked to human pain. Patients bearing an N855S gain-of-function mutation in this channel are afflicted with Familial Episodic Pain Disorder, a condition associated with upper body pain that can be triggered by stressful stimuli such as fatigue, cold, or fasting [175]. Reactive oxygen species acting at TRPA1 may also be responsible for the augmented cutaneous sensitivity to ambient light in patients with porphyria, a potential contributor to pain in that condition [176].

#### 2.7.2. TRPA1 and Itch

Multiple studies have shown that TRPA1 is also an important mediator of acute and chronic itch perception. Acute responses to non-histaminergic pruritogens (e.g., chloroquine, endothelin-1, compound 48/80) in the mouse are defective in the absence of TRPA1, owing to the positioning of this channel downstream of signaling by two pruritogen-sensing G protein-coupled receptors, MrgA3 and MrgC11 [177]. Endogenous pruritogens can also produce scratching behavior through TRPA1. For example, thymic stromal lymphopoietin (TSLP), which is released by keratinocytes in response to histaminergic signaling, activates neuronal TRPA1 downstream of the TSLP receptor [178]. Similarly, itch produced in response to bile acids, as might occur in the context of biliary obstruction, appears to be TRPA1 dependent, with the channel acting downstream of G protein-coupled bile receptors such as TGR5 [179]. The absence of TRPA1 has also been shown to reduce scratching behaviors in the acetone-ether-water mouse model of dry skin and chronic itch [180]. In addition, TRPA1 was upregulated in sensory neurons and TRPA1 antagonists reduced itch behavior in an interleukin 13-overexpressing transgenic model of atopic dermatitis [181]. Consistent with this observation, IL31 receptors were found to be colocalized with both TRPA1 and TRPV1 in sensory neurons, and genetic elimination of either channel reduced IL31-induced itch [48]. TRPA1 has also been shown to be an important contributor to the pruritis associated with contact dermatitis evoked by any of several different haptens [182]. This latter role might involve, among other mechanisms, direct action of TRPA1 by haptens, since 2,4-dinitrochlorobenzene and oxazolone, commonly used haptens for contact hypersensitivity experiments, have been shown to activate recombinant TRPA1 [182,183].

#### 2.7.3. TRPA1 and Inflammation

As a nonselective cation channel expressed in nociceptive and pruriceptive sensory neurons, TRPA1 is ideally suited to trigger the membrane depolarization necessary for the pain and itch sensations ascribed to it in the previous sections. However, evidence from a variety of studies indicates that the participation of TRPA1 in pathological processes related to itch and pain extends beyond its sensory function to include an active role in inflammation. For example, in the acetone-ether-water model of chronic dry skin [180] and in several different models of contact hypersensitivity [182], mice lacking TRPA1 showed reduced epidermal thickening, a suppressed cytokine response, and reductions in other hallmarks of inflammation. A similar proinflammatory role for TRPA1 has also been observed in other tissues, such as the lungs of mice with allergen-induced asthma [184].

#### 2.7.4. TRPA1 and Barrier Function

Although TRPA1 knockout mice have not been reported to exhibit any alterations in epidermal barrier function, there is pharmacological data to suggest that this channel can modulate barrier restoration. In hairless mice, cinnamaldehyde, mustard oil, and bradykinin all accelerated barrier recovery following tape stripping, and these effects could be blocked by a selective TRPA1 antagonist. Similarly, cooling of skin could accelerate barrier recovery, again in a manner inhibitable by the TRPA1 antagonist [185]. Given that TRPA1 and TRPV1 produce similar effects of cellular depolarization and calcium influx, it is interesting to compare these effects of TRPA1 activation with those described above for TRPV1, where channel activation appears to delay barrier recovery. Whether the apparently opposite consequences of activating these functionally similar channels relates to differential coupling to downstream signaling pathways, differential expression patterns, a complex consequence of channel activation and desensitization, or some other distinction remains to be determined.

#### 2.7.5. Loci of TRPA1 Action in Its Cutaneous Functions

Within neurons, the apparent sites at which TRPA1 might mediate its sensory functions include not only the peripheral terminals, where TRPA1 triggers action potential firing, but also their cell bodies and their central terminals in the spinal cord dorsal horn, where TRPA1 can modulate neurotransmitter release onto spinal circuits [186]. At least a component of the pro-inflammatory roles of TRPA1 in skin and in other tissues such as lung might also be attributable to its expression in sensory neurons. When stimulated, many of the sensory neurons expressing TRPA1 release pro-inflammatory neuropeptides such as substance P (SP) and calcitonin gene-related polypeptide (CGRP) into their target tissues, with consequent vasodilation and plasma extravasation [51]. Moreover, pharmacological or genetic blockade of SP signaling was shown to attenuate the inflammatory response associated with contact dermatitis [182]. However, the relationship between neuronal peptide release and inflammation is not entirely straightforward, since the neurogenic release of SP and CGRP has been shown to paradoxically suppress recruitment of immune cells to sites of bacterial infection [187] and since these neurons might also release anti-inflammatory peptides such as somatostatin [188].

As with many other TRP channels, TRPA1 expression in the skin is not confined to neurons. For example, expression of this channel has also been observed in cutaneous mast cells, where it is upregulated in the interleukin 13 overexpression mouse model of atopic dermatitis [181].

There is also evidence for TRPA1 expression in keratinocytes, although again this finding might differ between species. While it was originally reported that TRPA1 is expressed directly in mouse keratinocytes, a recent study, called this finding into question [189]. Although this leaves the function of TRPA1 in mouse keratinocytes unclear, transient expression of TRPA1 in keratin 14-lineage cells and consequent roles in the establishment of mechanosensory circuits cannot be excluded as a possibility. By comparison, TRPA1 expression in human keratinocytes is more strongly supported. In human scalp skin-derived cultures, TRPA1 mRNA and a TRPA1-like Western blot band were observed in primary keratinocytes. TRPA1-like immunoreactivity was also observed in basal epidermal and hair follicle keratinocytes in skin biopsies [181].

TRPA1 mRNA and protein were also detected in human scalp skin-derived melanocytes, and fibroblasts [181]. Moreover, by means of its responsiveness to ROS, TRPA1 mediates the earliest component of the bimodal hyperpigmentation response of melanocytes to Ultraviolet A (UVA) irradiation [190]. Several human melanoma-derived cell lines were also shown to express TRPA1 and to exhibit calcium responses to TRPA1 agonists that could be suppressed by a TRPA1 selective antagonist. However, in the same study, TRPA1 agonist-induced changes in cellular proliferation were insensitive to this same antagonist, potentially arguing against a clear role for this channel as a target of melanoma therapy [191]. Given these findings, additional tools and experiments will be required to precisely define the sites at which TRPA1 produces its effects on skin biology and the mechanisms by which it does so.

### 2.8. TRPM1 and Skin

#### 2.8.1. TRPM1 and Melanocytes

TRPM1 is prominently expressed in melanocytes, where it appears to participate in the generation of the photoprotective pigment, melanin, presumably by increasing intracellular Ca^2+^ levels [192]. Regulation of TRPM1 in melanocytes occurs at multiple levels, including transcription of TRPM1 mRNA [193], generation of different TRPM1 splice variants [194], TRPM1 activation downstream of G protein-coupled receptors such as metabotropic glutamate receptor 6 [195], and, possibly, miRNA regulation of TRPM1 mRNA stability or translation [196]. An early clue that TRPM1 was functionally important in melanogenesis came from the observation that mutations in the TRPM1 gene with incomplete dominance results in so-called “leopard spotting”, the patchy loss of pigmentation in the skin of Apalloosa horses [197]. In humans, TRPM1 expression levels are correlated with melanin content, with greater levels of TRPM1 in populations with more heavily pigmented skin [198]. The contribution of TRPM1 to pigmentation might be relevant to the pathogenesis of human vitiligo. Lesional skin in patients with either localized or generalized vitiligo shows significantly reduced levels of TRPM1 mRNA expression [196]. Within melanocytic nevii, TRPM1 expression drops as the melanocytes progress towards a less-differentiated melanoblast phenotype that does not produce melanin [194]. TRPM1 might also be involved in UV induced changes in skin pigmentation, though this link has not been explicitly confirmed [192]. One barrier to better defining the biological functions of TRPM1 in this process is that mice lack epidermal melanocytes, and thus do not exhibit UVB induced pigmentation changes. However, experiments involving humanized mouse skin might help overcome this limitation [199].

#### 2.8.2. TRPM1 and Melanoma

TRPM1 has also long been linked to melanoma, where reduced levels of TRPM1 expression and the appearance of alternatively spliced forms of TRPM1 mRNA are correlated with a less differentiated and more malignant phenotype [192,194]. While it is possible that TRPM1 activity, per se, contributes to melanoma progression and invasiveness, a more likely mechanism arises from the fact that miRNA 211, a tumor suppressor miRNA, is encoded in one of the introns of the TRPM1 gene and its transcription is co-regulated with that of TRPM1 [200]. 

### 2.9. TRPM2 and Skin

#### 2.9.1. TRPM2 and Cutaneous Pain Sensation

TRPM2 is a widely expressed channel that can be activated by two types of stimuli: reactive oxygen species (e.g., H_2_O_2_) and warm temperatures. It was recently demonstrated that this channel is required for normal behavioral responses of mice to innocuous warm temperatures [201]. Consistent with this requirement, TRPM2 is expressed in a subpopulation of peripheral sensory neurons, and genetic elimination of TRPM2 results in an apparent reduction in the proportion of sensory neurons responsive to warm temperatures but not to other chemical agonists of heat-sensitive TRP channels. Curiously, however, the proportion of cells that express TRPM2 mRNA is far greater than that responsive to warmth. The basis of this discrepancy is unclear, but it might reflect inefficient translation or trafficking of TRPM2 protein or a requirement for other components for full thermal responsiveness.

#### 2.9.2. TRPM2 and Melanoma

TRPM2 is also expressed in melanocytes and its activity can be suppressed by an endogenous dominant negative splice variant, TRPM2-TE. This splice variant is upregulated in melanoma, and either prevention of its synthesis or exogenous overexpression of full-length TRPM2 renders melanoma cells more susceptible to apoptosis and necrosis [194].

### 2.10. TRPM3 and Skin

#### TRPM3 and Cutaneous Pain Sensation

TRPM3 is expressed in a wide range of cell types, including a subpopulation of small to medium diameter sensory neurons. Like several other TRP channels expressed in this latter cell type, TRPM3 can be activated by painfully hot temperatures. It can alternatively be activated by chemical agonists, including the neurosteroid, pregnenolone sulfate and sphingolipids such as d-erythro-sphingosine [202]. Another curious feature of TRPM3 is that, in addition to a conventional nonselective cation pore, when this channel is stimulated with a combination of PS and clotrimazole, it exhibits a second, parallel ion permeation path, selective for small monovalent cations [203]. The physiological significance of this secondary conduction pathway, which resembles the “omega current” observed in some mutant voltage-gated sodium channels, has yet to be determined. Nevertheless, there is strong evidence supporting the physiological importance of TRPM3 to cutaneous sensory function. Neurons derived from mice lacking TRPM3 show defects in acute responsiveness to either PS or painful heat. Accordingly, these mice show behavioral deficits in response to these agonists, as well as defective heat hyperalgesia following inflammation with complete Freund’s adjuvant [202].

### 2.11. TRPM4 and Skin

#### TRPM4 and Lymphocytes

Although TRPM4 expression has not been demonstrated in either keratinocytes or sensory neurons, it is expressed in various immune cell populations, including T lymphocytes. Comparison of TH2 and TH1 cells has revealed a higher TRPM4 expression level in the former cells [18]. Inhibition of TRPM4 expression in TH2 cells leads to an elevation in intracellular calcium levels, with associated alterations in cytokine release profile. This seemingly paradoxical finding likely stems from the fact that TRPM4 is monovalent cation-selective, and activation of this channel depolarizes the membrane without associated calcium flux. That depolarization, moreover, reduces the electrochemical driving force for calcium influx through Orai channels. Thus, TRPM4 is poised to regulate T cell polarization and inflammatory responses in the skin.

### 2.12. TRPM7 and Skin

#### TRPM7 and Melanocytes

Although TRPM7 has not been studied extensively in mammalian skin, studies in zebrafish illustrate an important role for this channel in melanocytes. Melanocytes in mutant zebrafish lacking TRPM7 undergo death by necrosis [204]. This phenotype could be ameliorated by prevention of melanin synthesis, suggesting that the importance of TRPM7 is to facilitate chemical detoxification of intermediates of melanin synthesis. 

### 2.13. TRPM8 and Skin

#### 2.13.1. TRPM8 and Cutaneous Cold and Pain Sensation

TRPM8 was originally identified as a protein differentially expressed in prostate cancer cell lines. However, it was subsequently “rediscovered” as a receptor for cold-mimetic compounds such as menthol and icilin, as well as a channel that could be gated by mildly cold temperatures, themselves. TRPM8 is robustly expressed by a subpopulation of primary sensory neurons that are responsive to these same stimuli. Consistent with this expression pattern, genetic knockout experiments have shown convincingly that TRPM8 is required for normal responsiveness of sensory neurons to mild cold and, even more strikingly, for mouse behavioral avoidance of uncomfortably cool temperatures [205,206,207]. By comparison, the relationship of TRPM8 to pain is more complex. On the one hand, TRPM8 expression in a population of nociceptive neurons that also express TRPV1 appears to contribute to hypersensitivity to cold under conditions of inflammation or nerve injury. On the other hand, behavioral avoidance of intense cold is largely unaffected in TRPM8 knockout mice and under certain circumstances, TRPM8 stimulation (presumably in TRPM8-only neurons) actually suppresses pain sensitivity [208]. This cross-modal suppression is analogous to that observed between cold and itch perception [209], and is probably mediated by central nervous system circuits. At the same time, within sensory neurons, activation of mu opiate receptors leads to internalization of TRPM8 protein, providing a possible mechanism for opioid induced cold analgesia [210].

Another, recently identified TRPM8 activator is testosterone [211,212]. Androgen-dependent upregulation of TRPM8 gene expression has long been recognized in androgen-dependent prostate tumors [213]. However, in addition to its conventional transcription-mediated mechanisms of action, testosterone appears to activate TRPM8 by directly binding to the extracellular domain of the channel. In a recent human psychophysical study, testosterone application to the skin produced a cooling sensation, followed by a mild stinging sensation. This dual response was greater in female subjects than in males. Based on these findings, the authors speculated that testosterone acts as a natural cold-mimetic agent, but that high endogenous testosterone levels in males might desensitize TRPM8 over time. If true, this might account, in part, for the enhanced sensitivity of females to cold. Further experiments will be necessary to evaluate this provocative notion.

#### 2.13.2. TRPM8 and Epidermal Homeostasis 

With respect to non-thermosensory cutaneous functions for TRPM8, the earliest studies of TRPM8 knockout mice yielded no obvious epidermal phenotypes [205,206,207]. However, according to a more recent study [214], this lack of phenotype might have been a consequence of the specific strategies used to generate those mice, since those early strategies were based on disrupting exons encoding the TRPM8 amino terminus. Full-length TRPM8, which contains this domain, is not prominently expressed in keratinocytes. However, the authors of this recent study found that both human and mouse keratinocytes express an N terminally truncated TRPM8 splice variant lacking the N terminus and the first two transmembrane domains. They named this splice variant epithelial TRPM8 (eTRPM8). Genetically ablating the pore region shared by all TRPM8 isoforms, including eTRPM8, resulted in mice with an epidermal homeostasis phenotype. Specifically, these “pan-TRPM8 knockouts” exhibited a reduced proliferative cell number in the basal epidermis, an increase in superficial, late stage epidermal differentiation, and a reduction in the thickness of the stratum corneum. Unlike full-length TRPM8, eTRPM8 protein is confined to the endoplasmic reticulum, where it functions as a calcium release channel that facilitates elevations in calcium within adjacent mitochondria in response to canonical TRPM8 stimuli such as icilin, menthol, or cold. The authors of this study outlined two potential mechanisms by which this response might alter keratinocyte biology. First, mitochondrial calcium influx enhances synthesis of adenosine triphosphate, which when released from keratinocytes might alter their proliferation and differentiation in an autocrine manner. Second, in response to mildly cold stimuli, eTRPM8 augments production of superoxide, another candidate modulator of epidermal proliferation/differentiation. While these are certainly plausible explanations for the pan-TRPM8 knockout epidermal phenotype, additional cell type-specific knockout experiments will be required to determine whether the phenotype is truly a keratinocyte-autonomous phenomenon, or whether neuronal TRPM8 is also a contributor.

Other studies have provided additional links between TRPM8 and epidermal homeostasis. For example, TRPM8 agonists, like TRPA1 agonists, have been shown to accelerate barrier recovery following tape stripping of the skin and to reduce the epithelial proliferation response of the skin to barrier disruption [215]. It has also been reported that either chemical or thermal TRPM8 activation can reduce PGE2 release from human keratinocytes in response to UVB irradiation [216]. Whether these effects are mediated by full-length TRPM8 and/or eTRPM8 has not yet been established. 

#### 2.13.3. TRPM8 and Melanoma

TRPM8 expression has been observed in human melanoma cells, where its activation results in elevations in intracellular calcium and reduced cell viability [217,218]. This finding suggests that TRPM8 agonists might serve as candidate therapeutics for melanoma.

### 2.14. TRPML3 and Skin 

#### TRPML3 and Melanocytes

Members of the TRP-mucolipin (TRPML) channel subfamily are most notable for their functions not at the plasma membrane, but rather within intracellular organelles. One member of this family, TRPML3, has been shown to be especially important for normal melanocyte differentiation [219]. TRPML3 is highly expressed in healthy melanocytes. However, a gain-of-function mutation in the TRPML3 gene is responsible for the phenotypic traits of varitint-waddler mutant mice, which exhibit a combination of a vestibular defect and coat pigmentation defects. The pigmentation defect appears to result from the constitutive elevation of intracellular calcium in the melanocytes of these animals, and their subsequent cell death.

## 3. Conclusions 

In summary, numerous TRP channels are expressed in the cell types that constitute the skin, and they contribute in multiple ways to skin physiology under healthy conditions and to the pathological changes that occur in the setting of skin disease. Consequently, strategies aimed at this functionally diverse family of ion channels might provide useful means of combatting disorders of cutaneous homeostasis, sensation, and neoplasia.
